# Growth Hormone Therapy in Adults with Prader-Willi Syndrome

**DOI:** 10.3390/diseases3020056

**Published:** 2015-04-16

**Authors:** Karen S. Vogt, Jill E. Emerick

**Affiliations:** Division of Endocrinology, Department of Pediatrics, Walter Reed National Military Medical Center, Bethesda, 20889, MD, USA; E-Mail: Jill.E.Emerick.mil@mail.mil

**Keywords:** Prader-Willi, adult, growth hormone, body composition

## Abstract

Prader-Willi syndrome (PWS) is characterized by hyperphagia, obesity if food intake is not strictly controlled, abnormal body composition with decreased lean body mass and increased fat mass, decreased basal metabolic rate, short stature, low muscle tone, cognitive disability, and hypogonadism. In addition to improvements in linear growth, the benefits of growth hormone therapy on body composition and motor function in children with PWS are well established. Evidence is now emerging on the benefits of growth hormone therapy in adults with PWS. This review summarizes the current literature on growth hormone status and the use of growth hormone therapy in adults with PWS. The benefits of growth hormone therapy on body composition, muscle strength, exercise capacity, certain measures of sleep-disordered breathing, metabolic parameters, quality of life, and cognition are covered in detail along with potential adverse effects and guidelines for initiating and monitoring therapy.

## 1. Introduction

Prader-Willi syndrome (PWS) is a complex genetic disorder caused by lack of expression of genes on the paternally inherited chromosome 15q11.2-q13. Approximately 70% are due to deletion of this region, 25% are due to maternal uniparental disomy (UPD) of chromosome 15, and most of the remaining 5% are due to an imprinting center defect [1]. In adulthood, clinical manifestations include hyperphagia, obesity if food intake is not strictly controlled, decreased basal metabolic rate, sleep-disordered breathing, cognitive disability, short stature, and hypogonadism. The following are also characteristic of the syndrome: low muscle tone and abnormal body composition with decreased lean body mass and increased fat mass. Hypothalamic dysfunction has been implicated in many manifestations of this syndrome, including growth hormone (GH) insufficiency. The benefits of recombinant growth hormone therapy (hGH) on body composition and motor function, in addition to linear growth, in children with PWS are well established [2,3]. Evidence is emerging on the benefits of growth hormone therapy in adults with PWS as well. This review summarizes the current literature on growth hormone status and use of hGH in adults with PWS. We discuss the benefits of growth hormone therapy on body composition, muscle strength, exercise capacity, certain measures of sleep-disordered breathing, metabolic parameters, quality of life, and cognition. We also discuss potential adverse effects and guidelines for initiating and monitoring therapy.

## 2. Growth Hormone Status

Evidence of growth hormone insufficiency is present in the great majority of children with PWS [4,5]. There is also evidence of growth hormone insufficiency in many adults with PWS. Clinical evidence includes the abnormal body composition, which is also present in adults with growth hormone deficiency not affected by PWS [6]. Biochemically, adults with PWS have a diminished response to growth hormone provocative testing and lower insulin-like growth factor 1 (IGF-1) levels. Anatomically, anterior, but not posterior, pituitary size was found in one study to be significantly smaller in adults with PWS compared to healthy controls [7]. Another study found differences in cardiac anatomy and function, specifically decreased left ventricular mass and end diastolic diameter, in adults with PWS compared to obese controls, consistent with the hypotrophic hypokinetic syndrome that is associated with growth hormone deficiency [8].

Compared to obese controls, adults with PWS have a significantly lower response to growth hormone provocative testing with growth hormone releasing hormone (GHRH) plus arginine, not only for peak growth hormone values, but also the more functional determination of instantaneous secretion rates [9,10]. In studies using GHRH + arginine, 8%–55% of adults with PWS met diagnostic criteria for severe growth hormone deficiency using body mass index (BMI)-specific cut-off values [7,9,11,12,13]. However, some have questioned the validity of the GHRH + arginine test in those with hypothalamic disorders, given that GHRH is of hypothalamic origin [14]. With arginine as a provocative agent, 67% met diagnostic criteria for severe growth hormone deficiency, and 40% met criteria with insulin-induced hypoglycemia in one study [15]. In several studies, those with the UPD genotype had a lower response to stimulation testing than those with deletion [9,13,16]. IGF-1 levels are significantly lower in adults with PWS compared to obese controls [9]. In several studies, 75%–91% of adults with PWS had IGF-1 levels below the normal range [9,13,15]. Insulin-like growth factor binding protein-3 (IGFBP-3) levels were below the 5th percentile in 27% of adults with PWS [15]. 

It is important to note however that sex steroids promote growth hormone secretion and affect IGF-1 levels [17]. Hypogonadism is prominent in PWS [1]. Less than half of the subjects in the above studies were receiving sex steroid replacement therapy, which may have affected growth hormone secretion and IGF-1 levels.

## 3. Benefits of Growth Hormone Therapy

Studies have shown benefits of growth hormone therapy in adults with PWS on body composition, muscle characteristics, motor function, exercise capacity, peak expiratory flow, certain measures of sleep-disordered breathing, metabolic parameters, quality of life, and cognition.

The Scandinavian study is the largest published randomized placebo-controlled trial of growth hormone therapy in adults with PWS. Forty-six patients were randomized to hGH therapy with 0.6 or 0.8 mg daily after the first month depending on body weight, or placebo for the first year. Following this period of randomized treatment, all patients were treated with hGH for a total of 2 years. Thirty-nine patients completed both phases of the study. Lean body mass (LBM) increased in the hGH treated group by 2.3 kg as measured by duel-energy x-ray absorptiometry (DXA), and did not change in the placebo group. Fat mass decreased in the hGH group but increased in the placebo group with a difference of 4.2 kg. Subcutaneous and visceral fat, measured by targeted computed tomography (CT), also decreased in the hGH treated group while it increased in the placebo group with the greatest difference seen in subcutaneous fat. These benefits on body composition were sustained after 2 years of hGH therapy. Body mass index (BMI) and waist circumference did not change significantly [18,19,20]. 

Other studies have shown similar effects of growth hormone therapy on body composition. Increases in LBM of 2.3–3.7 kg and decreases in percent fat mass (FM%) of 2.2%–3.8% have been reported after 1 year of hGH therapy at mean doses of 0.53–1 mg daily [21,22,23,24,25]. One cohort was followed for 4 years with hGH therapy and these benefits persisted [26]. Subcutaneous and visceral fat measured by targeted CT in this cohort decreased significantly after 1 year of hGH therapy and visceral fat remained markedly decreased after 4 years of therapy [22,26]. Another group reported an increase in LBM of 2.8 kg and decrease of 1.9% in FM% after 2 years of hGH therapy [27]. In a study of six adults with PWS who had been on growth hormone 0.2–0.5 mg daily for a median of 5.1 years, median LBM increased by 4.9 kg and median FM% decreased by 4.7%, with the changes occurring gradually over time [28]. Consistent with the Scandinavian study, there was no significant change in BMI in any of these studies. A recent study of 10 males with PWS treated with hGH at a mean dose of 0.35 mg/day in adulthood for a mean of 15.5 years reported maintenance of a higher fat free mass than fat mass in all [29].

Two studies investigated changes in BMI and body composition after growth hormone therapy was stopped. In one study of 11 adults with PWS, the improvements in LBM and FM% after 1 year of hGH therapy at a mean dose of about 1 mg/day reverted back to baseline after therapy was withdrawn during the 2nd year of the study [24]. In another study, BMI standard deviation score (SDS) increased significantly in the 2 years after cessation of growth hormone therapy in late adolescence [30].

Several studies have shown improvements with hGH in skeletal muscle characteristics and function. There was a significant increase in thigh muscle volume by targeted CT after 2 years of hGH therapy in the Scandinavian study [19]. Another group confirmed a significant increase in thigh muscle size by CT imaging in 15 adults with PWS after 2 years of hGH at a mean dose of 0.4 mg/day, as well as a significant increase in lumbar muscle size and change in muscle tissue attenuation, reflecting a decrease in lipid accumulation. Strength measured by handgrip dynamometer also increased significantly and correlated with the increase in lumbar muscle size [27]. 

Growth hormone therapy also positively affects exercise capacity in adults with PWS. With hGH therapy, participants in the above study were able to continue a treadmill exercise test significantly longer before reaching exhaustion [27]. The same group also reported a metabolic equivalents (MET) increase of 19% on a treadmill exercise test after 1 year of hGH therapy at a mean dose of 0.9 mg daily in 12 adults with PWS [25]. In another study, the number of bouts of moderate-vigorous physical activity per day increased significantly after 1 year of therapy at a mean dose of about 1 mg/day and reverted back to baseline after hGH was withdrawn during the 2nd year of the study [24]. Left ventricular mass improved after 1 year of hGH therapy at a mean dose of 0.96 mg/day without negative effects on cardiac function in a study of 13 adults with PWS [22]. Nine of these individuals were followed for 4 years on hGH and these cardiac benefits persisted [26]. Furthermore, peak expiratory flow improved by 33 l/min after 2 years of hGH therapy in the Scandinavian study [19].

A few studies showed improvements in metabolic parameters with hGH therapy. The inflammatory marker, C-reactive protein (CRP), decreased significantly in 2 studies after 1 year of hGH and remained decreased after 4 years of therapy in one cohort [22,26,27]. One study reported normalization of low baseline triiodothyronine (T3) levels with 1 year of hGH therapy [23]. In the Scandinavian study, low-density lipoprotein (LDL) cholesterol decreased significantly in the hGH *versus* placebo group with 1 year of therapy. However, there was no change in LDL levels after 2 years of therapy [18]. One study reported an increase in HDL cholesterol after 1 year of hGH therapy that decreased significantly when growth hormone was withdrawn during the 2nd year of the study [24]. Other studies, however, showed no change in lipids after 1 year of hGH [22,23,27].

Quality of life improved in adults with PWS after 2 years of growth hormone therapy as measured by two validated instruments, the 36-Items Short Form Health Survey (SF-36), and the Psychological General Well-Being Index (PGWBI). Improvements were seen in anxiety, depression, general health, and total scores, reported by both the PWS adults and their parents. Improvements reported by the PWS adults however were greater than those reported by their parents [31]. In another study, participants spontaneously reported improvements in psychological well-being after 1 year of hGH therapy [21]. An additional study reported improvements after 1 year of hGH in a 20-item behavioral assessment completed by the participants’ caregiver [23]. These studies were not controlled.

On neuropsychological testing during a 6 month double-blind randomized placebo-controlled trial of hGH, followed by a 12 month open label treatment period of all participants, significant improvements were noted at 6 and 18 months in the hGH treated group in mental speed and flexibility, and motor performance. No improvements were seen at 6 months in the untreated group. However, only 13 of the 19 patients in this study were genetically confirmed to have PWS [32].

Two years of hGH therapy in the Scandinavian study did not show improvement in bone mineral density [33]. Similarly, a more recent study showed no effect of long-term hGH on bone density but did show a positive effect on bone size and strength, measured by bending breaking resistance index [34].

## 4. Potential Adverse Effects of Growth Hormone Therapy 

Despite the significant benefits of hGH on body composition, quality of life, muscle strength, exercise capacity, and pulmonary function in adults with PWS, there remains concern for potential adverse effects of this treatment. Due to the obesity that is commonly present in adults with PWS, the concern for adverse metabolic sequelae from growth hormone therapy, especially impairment in glucose homeostasis, deserves careful exploration. Type 2 diabetes mellitus has been reported in 25% of adults with PWS with a mean age of onset of 20 years [35]. The association of hGH treatment with insulin resistance is well documented. Although the mechanism of action for this association is not well defined, changes in free fatty acid metabolism, non-physiologic levels of IGF-1, and chronic basal hyperinsulinemia represent potential causes [36]. 

A recent meta-analysis of seven studies evaluating efficacy and safety of hGH for at least 12 months in adults with PWS showed a small increase in fasting glucose, and trends toward higher fasting insulin and insulin resistance. No significant changes in hemoglobin A1c or development of diabetes mellitus were noted. The longest study included in this meta-analysis involved 6 patients treated over a mean of 5 years [37]. More recently, the Scandinavian study group investigated glucose homeostasis in relation to BMI in 39 adults with PWS before and after 12 months of hGH therapy. They found that hGH treatment at a mean dose 0.6 mg was associated with a small but significant increase in fasting glucose, 2 h glucose after 75 gm oral glucose tolerance test (OGTT), and homeostatic model assessment of insulin resistance (HOMA-IR), irrespective of BMI [38]. In a recent report of 10 adult males with PWS treated with hGH for a mean of 15.5 years, 3 (30%) developed diabetes while on therapy. All 3 also had significant associated weight gain [29]. Therefore, close monitoring of glucose homeostasis is warranted in adults with PWS treated with hGH and additional longer term studies are needed.

The most common adverse event associated with hGH therapy in adults with PWS is lower extremity edema. Edema occurred in approximately 15% of those studied [37]. The edema often resolved after decreasing the dose [39]. A patient in one study stopped the hGH due to myalgias associated with lower extremity edema [23]. Other adverse events noted in the largest group of PWS patients studied were headache and nausea. Of note, one patient in the Scandinavian study was reported to have carpal tunnel surgery during hGH therapy [20]. Significantly, in the recent meta-analysis study, sudden death was not reported as an adverse event in any of the 7 cohorts [37].

Individuals with PWS have a high incidence of both central and obstructive sleep apnea [40,41,42]. Factors contributing to sleep-disordered breathing include obesity, restrictive lung disease due to muscle weakness or scoliosis, reduced ventilatory response to hypercapnia, and hypoxia during sleep and wakefulness [43]. Therapy with hGH potentially worsens sleep-disordered breathing because increased IGF-1 levels lead to lymphoid hyperplasia [44,45]. Only one study of relatively short duration has examined the effects of hGH on sleep-related breathing disorders in adults with PWS. This study evaluated sleep related breathing disorders at baseline and 6 weeks after the initiation of hGH therapy in 15 children and 10 adults with PWS. Nine of the 10 adults showed improvement in the apnea/hypopnea index (AHI) and decrease in the frequency of central events. The one adult who showed worsening of the AHI and frequency of obstructive events had a concurrent respiratory infection and tonsillar hypertrophy [44]. Until further data more clearly define the relationship between hGH and sleep-disordered breathing in PWS, current guidelines recommend polysomnography, with treatment of significant findings, prior to starting hGH therapy, and repeat polysomnography within 3-6 months of hGH initiation [46]. In addition, it is prudent to titrate hGH dosing to keep IGF-1 levels in the normal range to minimize the possibility of lymphoid hyperplasia and, while on therapy screen for signs and symptoms of worsening sleep apnea.

## 5. Initiating and Monitoring Growth Hormone Therapy

After an extensive review of the literature, consensus guidelines for recombinant human growth hormone therapy in PWS were published in 2013 [46]. These guidelines are summarized in this section. For adults with genetically confirmed PWS, hGH therapy should be considered after an expert multidisciplinary evaluation. Guidelines recommend considering severe obesity (BMI > 40 kg/m^2^), uncontrolled diabetes mellitus, untreated severe obstructive sleep apnea, active cancer and psychosis as contraindications to hGH therapy. Cognitive impairment is not considered an adequate barrier to treatment and it is strongly stated that informed consent/assent should include a discussion of the known risks and benefits of therapy. Therapy with hGH should be used in conjunction with dietary, environmental and lifestyle interventions. It should not be viewed as a weight loss medication. Therapy should be continued as long as the benefits outweigh the risks.

Prior to initiating hGH therapy in adults with PWS, the following evaluation is recommended (Table 1). A baseline IGF-1 level should be measured and growth hormone provocative testing considered. Many regulatory agencies require provocative testing in order to diagnose growth hormone deficiency prior to allowing insurance coverage of hGH treatment for adults with PWS. However, many participants in the studies showing benefit of hGH therapy did not meet the cut-off definition of growth hormone deficiency. For example, only 14.6% of those in the Scandinavian study tested as growth hormone deficient with GHRH + arginine [11,20]. Other studies have reported improvements with hGH in body composition, muscle size, strength, and exercise capacity that were independent of growth hormone secretory status [25,27]. Therefore, relying on provocative testing results may deny treatment to those who would benefit.

It is also important to carefully document anthropometric status including weight, height, BMI, waist circumference, and skinfold thickness if possible. Screening for hypothyroidism and appropriate treatment is warranted. As central adrenal insufficiency has been associated with PWS, providers should consider evaluation of adrenal function on an individual basis [47,48,49]. Growth hormone inhibits 11β-hydroxysteroid dehydrogenase type 1 (11βHSD-1), resulting in less cortisone conversion to active cortisol. Thus any underlying adrenal insufficiency could be exacerbated by hGH initiation.

Metabolic status should be assessed with a hemoglobin A1c and fasting insulin and glucose levels. An oral glucose tolerance test should be considered for individuals with a strong family history of diabetes, acanthosis nigricans, or ethnic risk factors. For obese individuals, fasting lipids and liver transaminases (AST, ALT) should be assessed. Sleep oximetry is felt to be mandatory before starting hGH in all patients, preferably by polysomnographic evaluation. Other helpful evaluations include body composition by dual energy x-ray photon absorptiometry (DXA) or bioelectrical impedance, assessment of cognitive status, and assessment of motor function (consider physiotherapy or occupational therapy referral).

**Table 1:** Recommended evaluation prior to initiation of growth hormone therapy (hGH) in adults with Prader-Willi syndrome (PWS) [46].

**Anthropometrics**	Weight
	Height
	BMI
	Waist circumference
	Skin fold thickness if possible
**Endocrine Evaluation**	Baseline IGF-1
	Consider provocative GH testing
	Thyroid function assessment
	Consider screening for adrenal insufficiency
**Metabolic Evaluation**	Hemoglobin A1c
	Fasting glucose
	Fasting insulin
	Consider OGTT if other risk factors are present
**Obesity Related Evaluation**	Fasting lipids
	AST, ALT
	Blood Pressure
**Sleep Oximetry**	Polysomnography preferred
**Baseline Evaluations to Help Assess Benefit of Therapy**	Body composition (DXA or biochemical impedance)
	Cognitive status
	Motor function ( physiotherapy/occupational therapy referral)

Adults with PWS should be started on hGH therapy at a dose of 0.1–0.2 mg/day. Starting dose should take into consideration age, presence of edema, prior hGH exposure and sensitivity, and concomitant oral estrogen use. Once therapy is started, the hGH dose should be titrated based on clinical response with a goal of maintaining IGF-1 levels in the 0 to +2 SDS range for age and gender.

Once started on hGH, monitoring should address specific benefits and risks of the treatment and impact on other potential hormone deficiencies (Table 2). IGF-1 levels should be checked at least annually and as needed for dose titration. IGF-1 levels checked for titration purposes should occur no less than six weeks after the dose change [14]. We recommend checking thyroid function within 3–6 months of initiation of hGH therapy and at least annually. Continued monitoring of glucose homeostasis should occur at least annually. For obese patients, routine monitoring of obesity co-morbidities such as hypertension, hyperlipidemia, and hepatic steatosis should occur as recommended by obesity guidelines [50]. Monitoring for other side effects of growth hormone therapy should take place on a routine basis. Evaluation for lower extremity edema should be done at each visit as well as assessment for signs or symptoms of sleep apnea. As one of the most well established benefits of hGH therapy in adults with PWS is improved body composition, repeat evaluation by DXA or biochemical impedance should be performed every 2 years [14]. Formal measurements of cognitive function, quality of life or exercise tolerance should be considered on an individual basis.

**Table 2:** Recommended monitoring during hGH therapy in adults with PWS [46].

**Anthropometrics**	Every 3–6 months Weight BMI
	Every 6–12 months Waist circumference Skin fold thickness if possible
**Endocrine Monitoring**	IGF-1: annually and as indicated for dose titration
	Thyroid function: within 3–6 months and at least annually
**Metabolic Monitoring**	Annually Hemoglobin A1c Fasting glucose and insulin OGTT if high risk
**Obesity Related Monitoring**	Annually Blood pressure Fasting lipids Consider AST and ALT
**Side Effects**	Assess for lower extremity edema at every follow up visit
	Clinically screen for signs/symptoms of sleep apnea
**Benefits of Therapy**	Body composition assessment every 2 years
	Cognitive status evaluation: consider on individual basis
	Motor function evaluation: consider on individual basis

## 6. Conclusions

Current evidence supports that adults with PWS have lower baseline IGF-1 levels and a diminished response to GH provocative testing when compared to obese controls. Depending on the cohort studied and the provocative agents used, 8%–55% of adults with PWS met criteria for severe GH deficiency based on BMI specific cut-offs. Adults with PWS treated with hGH experience many benefits of therapy, even when GH provocative testing is normal. The most consistently reported benefit is a beneficial change in body composition with increased LBM and decreased fat mass. Other benefits include improved skeletal muscle function, increased exercise tolerance, increased left ventricular mass, increased peak expiratory flow, improvement in cognition and quality of life, and a decrease in the apnea/hypoxia index. Studies of relatively short term use of hGH in adults with PWS show relatively few side effects, with lower extremity edema and increased insulin resistance being the side effects most consistently reported. There is a dearth of information regarding the effects of hGH on sleep disordered breathing in adults with PWS and further study of this relationship is warranted. Monitoring for known side effects of therapy is crucial during treatment. Finally, given that most of the current studies are uncontrolled and of short duration, and the effect size of many reported benefits is small, further longer-term controlled studies on the benefits and risks of hGH therapy in this patient population are necessary.

## Disclaimer

The views expressed in this publication are those of the authors and do not necessarily reflect the official policy or position of the Departments of the Army or Navy nor the US Government.
